# Induction of severe hypoxemia and low lung recruitability for the evaluation of therapeutic ventilation strategies: a translational model of combined surfactant-depletion and ventilator-induced lung injury

**DOI:** 10.1186/s40635-022-00456-5

**Published:** 2022-07-29

**Authors:** Emilia Boerger, Martin Russ, Philip von Platen, Mahdi Taher, Lea Hinken, Anake Pomprapa, Rainer Koebrich, Frank Konietschke, Jan Adriaan Graw, Burkhard Lachmann, Wolfgang Braun, Steffen Leonhardt, Philipp A. Pickerodt, Roland C. E. Francis

**Affiliations:** 1 Department of Anesthesiology and Intensive Care Medicine CCM/CVK, Charité – Universitätsmedizin Berlin, Corporate Member of Freie Universität Berlin and Humboldt-Universität zu Berlin, Augustenburger Platz 1, 13351 Berlin, Germany; 2 Institute of Biometry and Clinical Epidemiology, Charité – Universitätsmedizin Berlin, Corporate Member of Freie Universität Berlin and Humboldt-Universität zu Berlin, Charitéplatz 1, 10117 Berlin, Germany; 3 Fritz Stephan GmbH, Kirchstr. 19, 56412 Gackenbach, Germany; 4 EKU Elektronik GmbH, Am Sportplatz, 56291 Leiningen, Germany; 5 Chair for Medical Information Technology, Helmholtz-Institute for Biomedical Engineering, RWTH Aachen University, 52074 Aachen, Germany

**Keywords:** Acute lung injury, Acute respiratory distress syndrome, Surfactant depletion, Ventilator-induced lung injury, Recruitment maneuver, Mechanical power, Closed-loop ventilation

## Abstract

**Background:**

Models of hypoxemic lung injury caused by lavage-induced pulmonary surfactant depletion are prone to prompt recovery of blood oxygenation following recruitment maneuvers and have limited translational validity. We hypothesized that addition of injurious ventilation following surfactant-depletion creates a model of the acute respiratory distress syndrome (ARDS) with persistently low recruitability and higher levels of titrated “best” positive end-expiratory pressure (PEEP) during protective ventilation.

**Methods:**

Two types of porcine lung injury were induced by lung lavage and 3 h of either protective or injurious ventilation, followed by 3 h of protective ventilation (*N* = 6 per group). Recruitment maneuvers (RM) and decremental PEEP trials comparing oxygenation versus dynamic compliance were performed after lavage and at 3 h intervals of ventilation. Pulmonary gas exchange function, respiratory mechanics, and ventilator-derived parameters were assessed after each RM to map the course of injury severity and recruitability.

**Results:**

Lung lavage impaired respiratory system compliance (*C*_rs_) and produced arterial oxygen tensions (P_a_O_2_) of 84±13 and 80±15 (F_I_O_2_ = 1.0) with prompt increase after RM to 270–395 mmHg in both groups. After subsequent 3 h of either protective or injurious ventilation, P_a_O_2_/F_I_O_2_ was 104±26 vs. 154±123 and increased to 369±132 vs. 167±87 mmHg in response to RM, respectively. After additional 3 h of protective ventilation, P_a_O_2_/F_I_O_2_ was 120±15 vs. 128±37 and increased to 470±68 vs. 185±129 mmHg in response to RM, respectively. Subsequently, decremental PEEP titration revealed that *C*_rs_ peaked at 36 ± 10 vs. 25 ± 5 ml/cm H_2_O with PEEP of 12 vs. 16 cmH_2_O, and P_a_O_2_/F_I_O_2_ peaked at 563 ± 83 vs. 334 ± 148 mm Hg with PEEP of 16 vs. 22 cmH_2_O in the protective vs. injurious ventilation groups, respectively. The large disparity of recruitability between groups was not reflected in the *C*_rs_ nor the magnitude of mechanical power present after injurious ventilation, once protective ventilation was resumed.

**Conclusion:**

Addition of transitory injurious ventilation after lung lavage causes prolonged acute lung injury with diffuse alveolar damage and low recruitability yielding high titrated PEEP levels. Mimicking lung mechanical and functional characteristics of ARDS, this porcine model rectifies the constraints of single-hit lavage models and may enhance the translation of experimental research on mechanical ventilation strategies.

**Supplementary Information:**

The online version contains supplementary material available at 10.1186/s40635-022-00456-5.

## Background

The acute respiratory distress syndrome (ARDS) is a leading cause of mortality in perioperative and critically ill patients [1, 2]. Preclinical research that aims at identifying novel and targeted treatment approaches builds on various different experimental models of lung injury. However, none of these models can reflect all the pathological features and clinical characteristics of ARDS [3–5].

For example, pulmonary lavage-induced surfactant depletion is a widely used porcine model of acute hypoxemic lung failure. However, rapid recovery of respiratory system compliance (*C*_rs_) and blood oxygenation occur with this model after alveolar recruitment [6–9], indicating that the gas exchange abnormalities reflect collapsed alveoli with otherwise intact alveolar walls and high recruitability. In contrast, the clinical presentation of ARDS in patients typically includes alveolar injury and non-aerated lung tissue with low *C*_rs_ and limited recruitability [10]. Recruitability expresses the gain of aerated lung tissue following a recruitment maneuver. In patients and laboratory animals alike, high tidal volumes can lead to the inspiratory re-opening of previously atelectatic alveoli and thereby improve oxygenation. These alveoli may, however, re-collapse at end-expiration if PEEP is below the alveolar closing pressure. Such cyclic recruitment/derecruitment is associated with high shear stress contributing to pulmonary inflammation and ventilator-induced lung injury (VILI), which in turn can aggravate ARDS. Based on these considerations, experimental models of acute lung injury with prompt improvement of *C*_rs_ and oxygenation following recruitment maneuvers may be of limited translational validity. Several research groups have combined pulmonary lavage-induced surfactant depletion with mechanical ventilation of high tidal volumes and low PEEP to produce a type of lung injury with better similarity to human ARDS [11–13]. However, in many studies it is difficult to determine the extent to which the lung injury was caused by the lavage-induced surfactant depletion, the mechanical ventilation, or both.

In this study, we tested the effect of recruitment maneuvers on *C*_rs_ and oxygenation in a porcine model of lavage-induced surfactant depletion with and without the addition of injurious mechanical ventilation. Decremental PEEP trials were implemented to assess if the PEEP at best *C*_rs_ versus the PEEP at best oxygenation would differ between the two models. In addition, we implemented a software algorithm into the ventilator for the continuous automatic calculation of the mechanical power (*M*_*P*_) in order to estimate the diagnostic value of *M*_*P*_ as an indicator of acute lung injury during protective ventilation.

## Methods

This study was approved by the institutional animal welfare officer and the state authority for the care and use of animals (Tierversuchskommission, Landesamt für Gesundheit und Soziales, Berlin, Germany; approval number G 0229/18). Twelve male German Landrace pigs (bodyweight (BW) 46 ± 3 kg, mean ± SD) were housed under enriched standardized environmental conditions (22 °C, 12 h light–dark cycle) in groups of two to five at the animal facility of Charité—Universitätsmedizin Berlin (FEM Forschungseinrichtung für Experimentelle Medizin, Tierhaltung). Animals were fed standard chow (Complete feed for pigs, item nbr. 516040, AGRAVIS Ost GmbH, Germany) and fasted for 12 h with free access to water and hay before each experiment.

### Instrumentation

After intramuscular premedication with azaperone (3 mg/kg BW), atropine (0.03 mg/kg BW), ketamine (25 mg/kg BW) and xylazine (3.5 mg/kg BW) at 8:00 AM, a custom-made face mask was fitted on the snout for insufflation of ~ 10 L/min oxygen. A venous cannula (18–20 G, Braunüle®, B. Braun, Germany) was then inserted into an ear vein, a bolus of 500 ml of a balanced crystalloid solution (Sterofundin®, B. Braun, Germany) was administered and followed by continuous infusion of 4 ml/kg/h. Fractional boluses of 100 µg fentanyl were injected (no more than 1000 µg total) to titrate analgesia and sedation. The face mask was connected to the ventilator (EVE®, Fritz Stephan GmbH, Germany, CPAP-ASB mode) to provide assisted spontaneous breathing with PEEP 2 cmH_2_O, pressure support 5 cmH_2_O, and flow trigger 2 L/min. Subsequently, animals were placed supine for surgical tracheostomy after local anesthesia with infiltration of at least 10 mL of 2% lidocaine. After insertion of the tracheal cannula (9.0 ID tube; Mallinckrodt™; Covidien Deutschland GmbH, Neustadt, Germany), 5–10 mg/kg BW of propofol were injected. Then, the ventilator was connected to initiate pressure-controlled ventilation with volume guarantee (DUOPAP, EVE®, Fritz Stephan GmbH, Germany) and the following settings: fraction of inspired oxygen (F_I_O_2_) 1.0, tidal volume (V_T_) 9 ml/kg BW, PEEP 7 cmH_2_O, inspiratory to expiratory time ratio (I:E) 1:2 and respiratory rate (RR) adjusted to achieve an end-expiratory partial pressure of carbon dioxide (P_a_CO_2_) of 35–40 mmHg. Anesthesia was maintained with a continuous infusion of thiopentone (20 mg/kg/h) and fentanyl (7 µg/kg/h). Invasive instrumentation was performed as described previously [14]. Briefly, vascular catheters were placed into the femoral artery, and through the external jugular vein into the superior vena cava and the pulmonary artery for continuous monitoring of arterial, central venous and pulmonary artery pressures, and for the quantification of thermodilution cardiac output (Vigilance®, Type VGS1, Baxter, Edwards Lifesciences LLC, Irvine, USA). Finally, a suprapubic urinary catheter (14 Ch Latex Balloon Catheter, Dahlhausen & Co. GmbH, Germany) was established. Hemodynamic and respiratory parameters were recorded continuously using Powerlab™ (Model 8/30) with software LabChart™ 7.3.7 Pro (ADInstruments GmbH, Specbach, Germany).

### Protocol

**Fig. 1:**
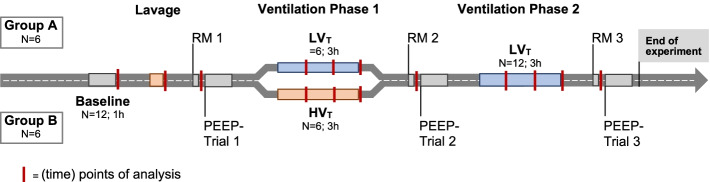
Experimental protocol. Anesthetized pigs underwent lavage-induced surfactant depletion followed by either low (LV_T_; *N* = 6) or high (HV_T_; *N* = 6) tidal volume ventilation during ventilation phase 1 (3 h). LV_T_ ventilation was resumed in ventilation phase 2 (3 h) in both groups. Recruitment maneuvers (RM) and PEEP trials were performed at three instances throughout the protocol to assess injury, recruitability, and “best PEEP”. For more details see “Methods” Section in the main text

Figure 1 depicts the experimental protocol. A 60 min baseline ventilation period was completed in pressure-controlled mode (DUOPAP) with low *V*_*T*_ of 6 ml/kg BW, PEEP 7 cmH_2_O, I:E 1:2, and RR adjusted to maintain P_a_CO_2_ at 37–45 mmHg. Next, surfactant-depletion was induced by a pulmonary lavage procedure (see below). Animals were then randomized (bag of lots) to undergo a 3 h period (ventilation phase 1) of either protective or injurious ventilation (*N* = 6 per group). Protective ventilation consisted of a low *V*_*T*_ (6 ml/kg) and tabular PEEP strategy following the rules of the ARDSNet (NIH NHLBI ARDS Network) protocol [15, 16]. To this end, an automated closed-loop mechanical ventilation algorithm adherent to the ARDSNet protocol was developed and used in this study to apply protective ventilation. Injurious ventilation consisted of a non-automated high *V*_*T*_ (17 ml/kg), low PEEP (2 cmH_2_O) strategy with RR of 12/min to prevent hypocapnia. These high tidal volumes produced a peak inspiratory pressure (PIP) of around 50 cmH_2_O, which corresponds to the upper pressure limit that was applied during the alveolar recruitment maneuvers in both groups (see below). The 3 h period of either protective or injurious ventilation was followed by additional 3 h of automated protective closed-loop ventilation (ventilation phase 2).

Recruitment maneuvers (RM) were performed at three instances during the course of the protocol: (i) after pulmonary lavage (RM1); (ii) after initial 3 h of protective vs. injurious ventilation (RM2); (iii) after final 3 h of protective ventilation (RM3). Pulmonary gas exchange function, respiratory mechanics, and ventilator-derived parameters were assessed after each RM to map the course of injury severity and pulmonary recruitability. Each RM was followed by a decremental PEEP trial to identify the PEEP associated with either the maximum *C*_rs_ or the maximum oxygenation (“best PEEP”). A cardioplegic bolus of potassium chloride was used to kill animals in deep anesthesia (bolus: 500 µg of fentanyl, 1000 mg thiopentone) at the end of the protocol. Lung tissue sample underwent histopathological assessment for signs of lung injury.

#### Pulmonary lavage procedure

Lavage-induced surfactant depletion was performed to induce acute lung injury (ALI) in all animals as described in detail elsewhere [9, 14]. Briefly, after neuromuscular blockade with pancuronium bromide (0.15 mg/kg BW i.v. bolus), repetitive lavages were performed using warm 0.9% saline until PaO_2_ at F_I_O_2_ 1.0 and PEEP 6 cmH_2_O was below 100 mmHg for 10 min.

#### Alveolar recruitment maneuver and decremental PEEP trial

A pressure-controlled ventilator mode was used for the recruitment maneuver and PEEP trial. First, a PEEP of 12 cmH_2_O was applied. The RR was then set to 20/min and Δ*P* (PIP − PEEP) to 20cmH_2_O. PEEP was then increased in steps of 2–4 cmH_2_O every 5 breaths until 24 cmH_2_O was reached. Then, Δ*P* was increased to apply a peak inspiratory pressure of 50 cmH_2_O at PEEP 24 cmH_2_O for 5 respiratory cycles. Immediately thereafter, PEEP 15 cmH_2_O and *V*_*T*_ 6 ml/kg BW were applied for 5 min, to allow the assessment of pulmonary function parameters (blood gas analysis), respiratory mechanics (dynamic *C*_rs_ and *M*_*P*_, and hemodynamics including cardiac output under standardized conditions. Next, for the purpose of equal volume history, the lungs were ventilated with *V*_*T*_ 10 ml/kg, PEEP 0 cmH_2_O, RR 10/min, I:E 1:1 for 10 respiratory cycles, followed by a brief disconnection from the ventilator (5 s). Then, a decremental PEEP titration was performed to identify the maximum P_a_O_2_ and maximum dynamic *C*_rs_ under these conditions: while keeping a constant Δ*P* of 14 cmH_2_O and RR of 20/min, PEEP was set at 12 cmH_2_O and was then increased to 24 cmH_2_O in steps of 4 cmH_2_O per every 5 respiratory cycles. A stepwise reduction of PEEP by 2 cmH_2_O, again with a constant Δ*P* of 14 cmH_2_O, was performed at intervals of 10 min. The titration was stopped when a minimum PEEP of 6 cmH_2_O or a P_a_O_2_/F_I_O_2_ ratio of less than 80 mmHg was reached. All measurements were performed at the end of each step.

#### Automated closed-loop mechanical ventilation system

We designed an automated closed-loop mechanical ventilation algorithm and ventilator system to apply protective ventilation adherent to the ARDSNet protocol [7, 8, 15]. Here, all components of the software, actuators and devices of the closed-loop physiological feedback algorithm were integrated into a mechanical ventilator system (EVE^®^, Fritz Stephan GmbH, Germany). The system outputs were designed to automatically adjust *V*_*T*_, PEEP, RR, I:E and F_I_O_2_ independently. System inputs were derived from an integrated capnograph and pulse oximeter for the continuous measurement of exhaled CO_2_ concentration and peripheral oxyhemoglobin saturation (SpO_2_), respectively. Briefly, an SpO_2_ target of 88–95% and the “higher PEEP/lower F_I_O_2_” table were used to adjust F_I_O_2_ and PEEP [16]. *V*_*T*_ of 6 ml/kg BW was delivered in a pressure-controlled mode (DUOPAP) with decelerating gas flow, and was automatically reduced to 5 or 4 ml/kg BW when PIP exceeded 30 cmH_2_O. The system received manual input of arterial pH (pH_a_) once every 30 min. At pH_a_ < 7.30 or > 7.45, the closed-loop system repetitively adjusted RR in steps of ± 5 /min, respectively. Maximum RR was limited at 35 /min.

#### Calculation of the mechanical power

Gattinoni et al. proposed *M*_*P*_ as a unified variable to encapsulate all ventilator-related causes of lung injury [17]. The original equation described by Gattinoni et al. is, however, only applicable to volume-controlled ventilation with constant inspiratory flow. Since this study used a ventilation mode with decelerating flow, the simplified equation for *M*_*P*_ proposed by Becher et al. for pressure-controlled ventilation was used:

$${M}_{P} = 0.098 \cdot RR \cdot {V}_{T} \cdot \left(\Delta P + PEEP\right).$$

This simple equation was shown to correlate well with the true *M*_*P*_ and to be acceptable for clinical purpose [18]. The other equations for *M*_*P*_ calculation during pressure-controlled ventilation proposed by Becher et al. and Van der Meijden et al. are computationally more costly, with only a small improvement in fit [18, 19].

### Statistical analysis

**Fig. 2:**
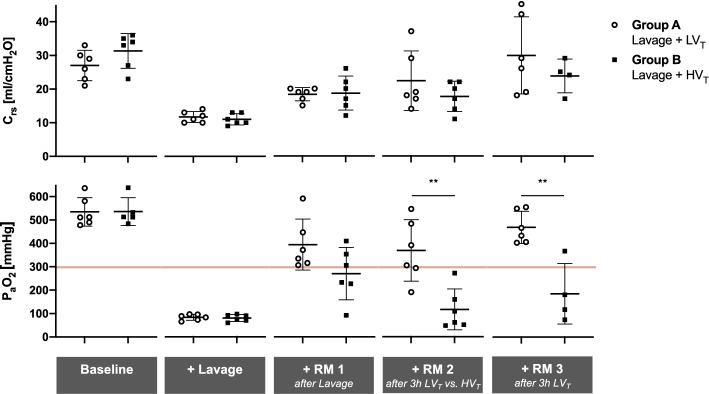
Respiratory mechanics and function. Dynamic compliance of the respiratory system (*C*_rs_) and arterial partial pressure of oxygenation (P_a_O_2_) were assessed in anesthetized pigs at baseline, after lavage-induced surfactant depletion, and after consecutive recruitment maneuvers (RM) at 3 h intervals. Group A, *N* = 6: continuous automated protective low tidal volume ventilation (LV_T_). Group B, *N* = 6: 3 h of injurious high tidal volume ventilation (HV_T_) prior to RM 2, resumption of protective low tidal volume ventilation for 3 h prior to RM 3. Two animals died after injurious HV_T_ and RM 2, i.e., Group B, *N* = 4 at RM 3. For experimental protocol, see Fig. 1. Scatter plots with horizontal (means) and error bars (SD). **p < 0.01, Mann–Whitney *U* test

**Fig. 3:**
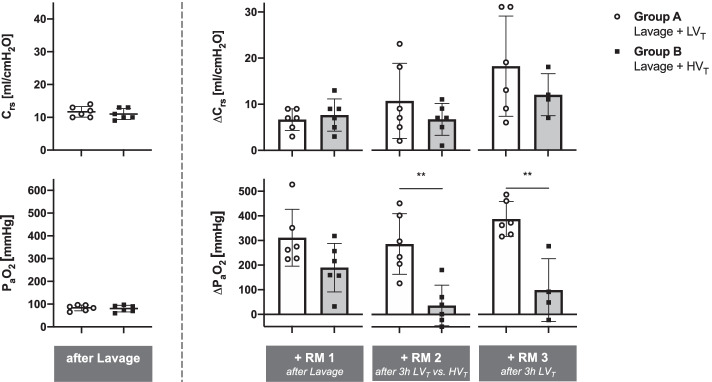
Ventilation-induced deviation from lavage-induced impairment of respiratory mechanics and function. Dynamic compliance of the respiratory system (*C*_rs_) and arterial partial pressure of oxygenation (P_a_O_2_) were assessed in anesthetized pigs after lavage-induced surfactant depletion, and after consecutive recruitment maneuvers (RM) at 3 h intervals. Figure depicts the relative deviation of *C*_rs_ and P_a_O_2_ (obtained after RM) from the respective values after initial lavage. Group A, *N* = 6: continuous automated protective low tidal volume ventilation (LV_T_). Group B, *N* = 6: 3 h of injurious high tidal volume ventilation (HV_T_) prior to RM 2, resumption of protective low tidal volume ventilation for 3 h prior to RM 3. Two animals died after injurious HV_T_ and RM 2, i.e., Group B, *N* = 4 at RM 3. For experimental protocol, see Fig. 1. Scatter/bar plots with horizontal (means) and error bars (SD). **p < 0.01, Mann–Whitney *U* test

**Fig. 4:**
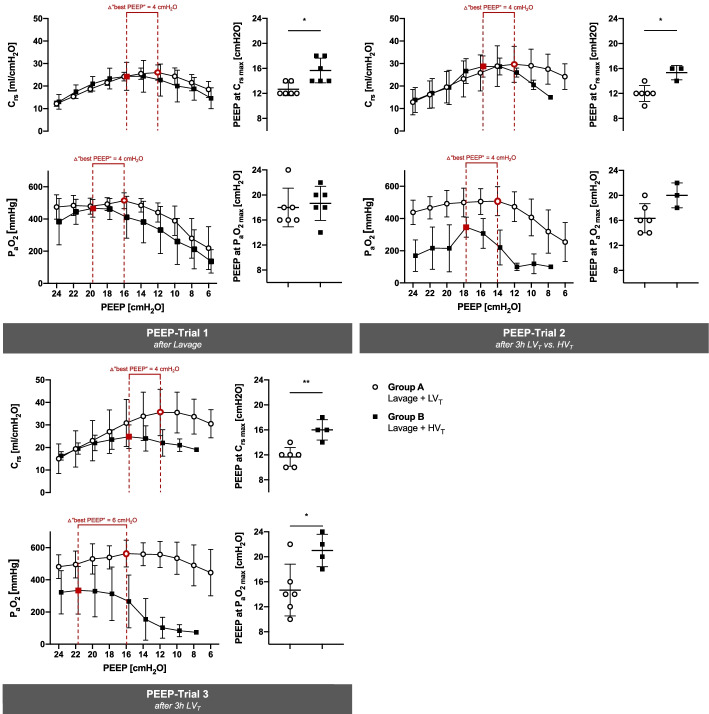
Decremental PEEP trials: identification of “best PEEP**”.** PEEP trials with decrements of 2 from 24 to 6 cmH_2_O were performed at three instances following a recruitment maneuver (RM) in anesthetized pigs which had undergone combined lavage-induced surfactant depletion and therapeutic ventilation. Group A, *N* = 6: continuous automated protective low tidal volume ventilation (LV_T_). Group B, *N* = 6: 3 h of injurious high tidal volume ventilation (HV_T_) prior to PEEP trial 2, resumption of protective low tidal volume ventilation for 3 h prior to PEEP trial 3. Two animals died after injurious HV_T_ and RM 2, i.e., Group B, *N* = 4 at PEEP trial 2 and 3. For experimental protocol, see Fig. 1 and “Methods” Section. Line graphs depict the group means of *C*_rs_ and P_a_O_2_ obtained at each PEEP level. PEEP values at the maximum mean *C*_rs_ and maximum mean P_a_O_2_ (red vertical dotted lines) are consistently disparate within each group and differ between groups. Adjacent scatter plots depict the PEEP values at maximum *C*_rs_ and P_a_O_2_ of each individual animal and the means ± SD thereof. *p < 0.05, **p < 0.01, Mann–Whitney *U* test

**Fig. 5:**
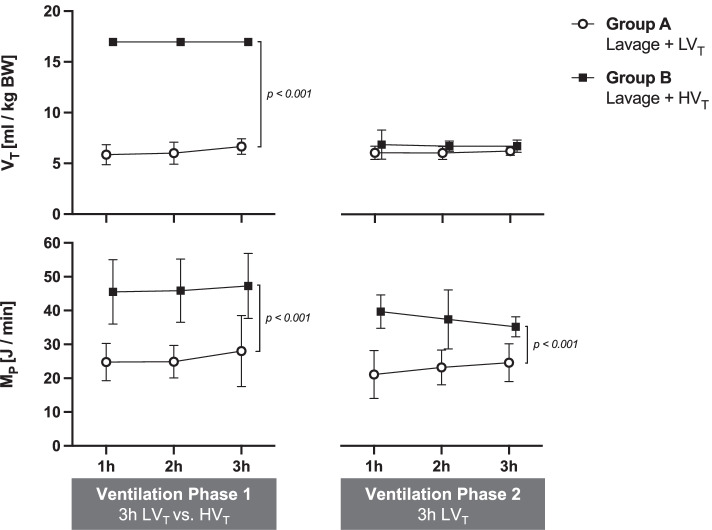
Tidal volume and mechanical power in combined lavage and ventilator-induced lung injury. Tidal volume (*V*_*T*_) and mechanical power (*M*_*P*_) of ventilation were assessed in anesthetized and surfactant-depleted pigs which underwent either an automated closed-loop protective low tidal volume (6 ml/kg BW LV_T_) and tabular PEEP ventilation strategy (group A) or non-automated injurious high tidal volume ventilation (HV_T_) with *V*_*T*_ of 17 ml/kg BW and PEEP 2 cmH_2_O (group B) during ventilation phase 1. Ventilation phase 2 consisted of automated LV_T_ ventilation in both groups (*N* = 6 each). Two animals died after injurious HV_T_ and RM 2, i.e., Group B, *N* = 4 during ventilation phase 2. For experimental protocol, see Fig. 1 and “Methods” Section. For calculation of the M_P_, the simplified equation proposed by Becher et al. for pressure-controlled ventilation is used (see “Methods” Section in the main text). Means ± SD; *p*-values indicate significant group effects and were calculated using two-way ANOVA-type *nparLD package* (see Table 2, associated relative treatment effects (RTE) see Additional file 1: Table S2a). For more statistical details refer to main text

For statistical computing, we used R version 4.0.3 (http://R-project.org) and GraphPad Prism software (GraphPad Software, Version 9.0.2, La Jolla, CA). All values are presented as means ± standard deviation (SD). Due to the rather small sample sizes, we used nonparametric ranking methods for making inference. Continuous variables of the two independent groups at discrete time points (e.g., after lavage or RM 1–3; in Figs. 2, 3, and 4) were compared using Mann–Whitney *U* test. Nonparametric longitudinal data analysis was performed with a two-way ANOVA-type statistic implemented in the *Nonparametric Analysis for Longitudinal Data (nparLD) package* [20]. The inter-group (group effect) and intra-group variables (time effect) were analyzed to identify main and interaction effects on the outcome parameters shown in all Table and Fig. 5. The associated treatment effect is the so-called relative treatment effect (RTE) which is the proportion of data from the entire data set being smaller (or equal) than the values in a subgroup or at an individual time point. Interpretation of RTEs is explained in more detail in Additional file 1: Table S1. Missing values in group B (lavage-induced surfactant depletion followed by injurious HV_T_ ventilation) during PEEP trials 2 and 3 are caused by the termination criterion (P_a_O_2_ < 80 mmHg). Consequently, the PEEP trials were excluded from the inferential longitudinal analysis. A two-tailed *p*-value < 0.05 was considered statistically significant. A series of sample size calculations (power 0.8, *α* = 0.05) based on different outcome parameters with variance estimated or known from previous experiments, suggested a group size between 6 and 10. Due to the ability to detect significant differences in the level of injury at interim analysis, we concluded experimentation after n = 6 per group.

## Results

### Effects of lavage-induced surfactant depletion

The pulmonary lavage procedure consistently produced severe hypoxemia and hypercapnia (both P_a_O_2_ and P_a_CO_2_ around 80 mmHg at F_I_O_2_ 1.0, *V*_*T*_ 6 ml/kg BW, PEEP 6 cmH_2_O), lower *C*_*rs*_, higher PIP, Δ*P* and mean pulmonary artery pressure (mPAP) compared to baseline, reflecting acute lung injury in both groups (Table 1, Fig. 2). A subsequent recruitment maneuver (RM1) ending with a *V*_*T*_ 6 ml/kg BW and PEEP 15 cmH_2_O, restored P_a_O_2_, *C*_*rs*_ and Δ*P* to a large extent (Table 1, Fig. 2).

**Table 1:** Respiratory and hemodynamic parameters

	Group	Baseline	+ Lavage	+ RM 1	+ RM 2	+ RM 3	*p* value		
				After Lavage	After 3 h LV_T_ vs HV_T_	After 3 h LV_T_	Group effect	Time effect	Group : Time
PIP [cmH_2_O]	A	20.8± 1.3	30.0 ± 2.8	31.2 ± 2.3	29.7 ± 4.9	27.2 ± 4.8	0.4881	< 0.001	0.1739
	B	20.2 ± 1.3	32.2 ± 5.3	32.0 ± 8.2	35.8 ± 6.9	27.5 ± 2.7			
PEEP [cmH_2_O]	A	7.2 ± 1.0	6.2 ± 0.4	15.0 ± 0.0	15.3 ± 0.5	15.3 ± 0.5	0.5759	< 0.001	0.0709
	B	7.3 ± 0.8	6.2 ± 0.4	15.3 ± 0.5	15.7 ± 0.5	15.0 ± 0.0			
△*P* [cmH_2_O]	A	13.7 ± 1.7	24.0 ± 2.8	16.1 ± 2.3	14.7 ± 4.9	12.1 ± 4.8	0.5983	< 0.001	0.2651
	B	12.8 ± 1.6	26.2 ± 5.3	19.9 ± 8.2	18.4 ± 3.9	12.5 ± 2.7			
*C*_rs_ [ml/cmH_2_O]	A	27.0 ± 4.5	11.7 ± 1.6	18.3 ± 2.0	22.3 ± 8.8	29.8 ± 11.4	0.6959	< 0.001	0.2601
	B	31.3 ± 5.2	11.0 ± 1.7	18.7 ± 5.1	17.7 ± 4.5	23.8 ± 5.0			
*M*_*P*_ [J/min]	A	20 ± 3	15 ± 3	17 ± 3	16 ± 2	15 ± 3	0.7976	< 0.001	0.4778
	B	19 ± 2	17 ± 3	17 ± 3	18 ± 6	14 ± 1			
P_a_O_2_ [mmHg]	A	535 ± 61	84 ± 13	395 ± 109	369 ± 132	470 ± 68	< 0.001	< 0.001	< 0.001
	B	536 ± 60	80 ± 15	270 ± 112	167 ± 87	185 ± 129			
P_a_CO_2_ [mmHg]	A	51 ± 5	80 ± 16	80 ± 13	97 ± 21	94 ± 21	0.1715	< 0.001	0.0882
	B	47 ± 6	84 ± 8	81 ± 9	103 ± 11	123 ± 10			
mPAP [mmHg]	A	17 ± 3	27 ± 8	23 ± 5	24 ± 6	22 ± 4	0.0417	< 0.001	0.0034
	B	15 ± 1	28 ± 2	23 ± 2	33 ± 6	30 ± 3			
PVR [dyn∙sec∙cm^−5^]	A	179 ± 68	/	257 ± 105	202 ± 77	153 ± 50	0.0256	< 0.001	0.1297
	B	159 ± 25	/	321 ± 92	369 ± 58	224 ± 113			
CO [L/min]	A	3.6 ± 1.3	/	4.4 ± 2.3	5.7 ± 2.6	6.3 ± 2.4	0.6719	< 0.001	0.1915
	B	4.2 ± 0.7	/	3.6 ± 1.2	5.3 ± 0.8	6.9 ± 1.0			

Respiratory mechanics, pulmonary function and hemodynamics were assessed in anesthetized pigs at baseline, after lavage-induced surfactant depletion, and after consecutive recruitment maneuvers (RM) at 3-h intervals. Group A, *N* = 6: continuous automated protective low tidal volume ventilation. Group B, *N* = 6: 3 h of injurious high tidal volume ventilation (HV_T_) before RM 2, resumption of protective low tidal volume ventilation (LV_T_) for 3 h before RM 3. Two animals died after injurious HV_T_ and RM 2, i.e., Group B, *N* = 4 at RM 3. For experimental protocol, see Fig. 1. Means ± SD; *p*-values for group effects (inter-group) and/or time effects (intra-group) were calculated using two-way ANOVA-type statistic using the *nparLD package*. For more statistical details, refer to main text. *PIP* peak inspiratory pressure, *PEEP* positive end-expiratory pressure, *ΔP* driving pressure, *C*_*rs*_ dynamic respiratory system compliance; *M*_*P*_ mechanical power, *P*_*a*_*O*_*2*_ arterial partial pressure of oxygen, *P*_*a*_*CO*_*2*_ arterial partial pressure of carbon dioxide, *mPAP* mean pulmonary artery pressure, *PVR* pulmonary vascular resistance, *CO* cardiac output

### Lung injury severity and recruitability after protective versus injurious ventilation

Subsequent to lavage and RM1, longitudinal analysis of pulmonary function and ventilator-derived parameters revealed significant group and time effects (Table 2): 3 h of protective vs. injurious ventilation (ventilation phase 1) with Δ*P* of 17 ± 3 vs. 49 ± 8 cmH_2_O, PEEP 11 ± 3 vs. 2 ± 0.5 cmH_2_O, PIP of 28 ± 5 vs. 51 ± 9 cmH_2_O, and M_P_ of 28 ± 11 vs. 47 ± 10 J/min, resulted in C_rs_ (20.8 ± 7.1 vs. 18.8 ± 3.3 ml/cmH_2_O) and P_a_O_2_/F_I_O_2_ (104 ± 26 vs. 154 ± 123 mmHg) of similar magnitude (Table 2). Following additional 3 h of protective ventilation (ventilation phase 2) with tabular PEEP of 11 ± 5 vs. 17 ± 3 cmH_2_O, ΔP was 15 ± 4 vs. 19 ± 3 cmH_2_O, PIP 26 ± 4 vs. 35 ± 4 cmH_2_O, and M_P_ 24.7 ± 5.6 vs. 35.3 ± 3.0 J/min. Again, *C*_rs_ (23.7 ± 7.1 vs. 21.5 ± 4.2 ml/cmH_2_O) and P_a_O_2_/F_I_O_2_ (120 ± 15 vs. 128 ± 37 mmHg) were similar in both groups that had previously received either protective or injurious ventilation, respectively (Table 2). Longitudinal analysis of injury severity was extended to the assessment of pulmonary compliance and oxygenation after RMs (Table 1, Figs. 2 and 3). In the group receiving pulmonary lavage followed by protective ventilation, RM 2 and 3 revealed instantaneous recruitability with complete restoration of *C*_rs_ and normalization of P_a_O_2_/F_I_O_2_ ratios (> 300 mmHg), but persistence of hypercapnia. In contrast, in the group that had received pulmonary lavage and injurious ventilation, RM 2 and 3 failed to restore *C*_rs_ and P_a_O_2_, leaving pulmonary function significantly impaired. Figure 3 depicts the relative deviation of *C*_rs_ and P_a_O_2_ (obtained after RM) from the respective values after initial lavage: a progressive gain in compliance and oxygenation can be observed along the time course in the protective ventilation group.

**Table 2:** Respiratory and hemodynamic parameters during low and high tidal volume ventilation

		Ventilation Phase 1—Group A: LV_T_, Group B: HV_T_						Ventilation Phase 2—Group A & B: LV_T_					
	Group	1 h	2 h	3 h	*p* value			1 h	2 h	3 h	*p* value		
					Group effect	Time effect	Group : Time				Group effect	Time effect	Group : Time
PIP [cmH_2_O]	A	29.8 ± 3.4	28.8 ± 4.2	28.2 ± 4.8	< 0.001	0.8864	0.3043	25.7 ± 2.4	26.2 ± 2.9	26.2 ± 3.5	< 0.001	0.5382	0.1304
	B	48.8 ± 7.5	50.2 ± 8.8	51.3 ± 8.6				38.5 ± 4.1	37.3 ± 8.1	35.3 ± 4.4			
PEEP [cmH_2_O]	A	12.2 ± 5.1	11.2 ± 5.1	10.8 ± 3.1	< 0.001	0.6574	0.6574	11.0 ± 3.8	11.7 ± 4.3	11.0 ± 4.6	< 0.001	0.4036	0.1401
	B	2.3 ± 0.5	2.3 ± 0.5	2.3 ± 0.5				20.0 ± 3.7	17.8 ± 3.5	16.5 ± 2.5			
△*P* [cmH_2_O]	A	17.7 ± 3.8	17.7 ± 4.1	17.3 ± 3.2	< 0.001	0.7429	0.3450	14.7 ± 4.1	14.5 ± 3.9	15.2 ± 3.6	0.0283	0.7331	0.9275
	B	46.5 ± 7.0	47.8 ± 8.4	49.0 ± 8.2				18.5 ± 1.0	19.5 ± 5.1	18.8 ± 2.6			
*V*_*T*_ [ml/kg BW]	A	5.9 ± 1.0	6.0 ± 1.1	6.7 ± 0.8	< 0.001	0.3351	0.3351	6.1 ± 0.7	6.0 ± 0.7	6.2 ± 0.4	0.1908	0.9432	0.8194
	B	17.0 ± 0.1	17.0 ± 0.1	17.0 ± 0.1				6.9 ± 1.4	6.7 ± 0.5	6.7 ± 0.6			
RR [x/min]	A	33 ± 4	33 ± 3	33 ± 3	< 0.001	0.3173	0.3173	30 ± 6	33 ± 3	34 ± 2	0.0540	0.1098	0.1098
	B	12 ± 0	12 ± 0	12 ± 0				35 ± 0	35 ± 0	35 ± 0			
*M*_*P*_ [J/min]	A	25 ± 1	25 ± 5	28 ± 11	< 0.001	0.0792	0.4419	21 ± 7	23 ± 5	25 ± 6	< 0.001	0.8837	0.1737
	B	46 ± 10	46 ± 9	47 ± 10				40 ± 5	38 ± 9	35 ± 3			
*C*_rs_ [ml/cmH_2_O]	A	17.8 ± 2.3	18.8 ± 4.4	20.8 ± 7.1	0.5463	0.7863	0.0423	23.5 ± 8.2	23.2 ± 7.3	23.7 ± 7.1	0.7851	0.4536	0.6960
	B	20.2 ± 1.8	18.7 ± 3.7	18.8 ± 3.3				20.3 ± 5.1	21.5 ± 4.7	21.5 ± 4.2			
P_a_O_2_ [mmHg]	A	117 ± 33	104 ± 29	104 ± 26	0.0567	< 0.001	0.0650	112 ± 20	111 ± 13	120 ± 15	0.1572	0.5621	0.0863
	B	246 ± 115	169 ± 108	154 ± 123				175 ± 67	172 ± 57	128 ± 37			
P_a_CO_2_ [mmHg]	A	87 ± 44	90 ± 51	72 ± 11	0.5409	0.4111	0.2052	85 ± 22	85 ± 20	84 ± 24	0.3105	0.9483	0.5646
	B	66 ± 8	70 ± 14	79 ± 23				98 ± 17	90 ± 23	92 ± 5			
mPAP [mmHg]	A	28 ± 4	28 ± 4	26 ± 7	0.5904	0.2583	0.0028	25 ± 5	25 ± 7	23 ± 5	0.2209	0.4519	0.2483
	B	26 ± 3	27 ± 3	33 ± 8				28 ± 2	27 ± 2	28 ± 4			
PVR [dyn∙sec∙cm^−5^]	A	369 ± 98	347 ± 111	318 ± 145	0.0104	0.9552	0.2871	241 ± 82	218 ± 103	190 ± 92	0.0346	0.1473	0.9471
	B	452 ± 143	479 ± 166	531 ± 132				352 ± 51	324 ± 107	285 ± 146			
CO [L/min]	A	4.5 ± 1.4	4.9 ± 1.4	5.4 ± 2.3	0.0621	0.1528	0.4519	6.0 ± 2.1	6.6 ± 1.2	6.8 ± 1.9	0.0843	0.3471	0.5706
	B	3.2 ± 1.1	3.4 ± 0.7	3.9 ± 0.9				4.7 ± 2.0	5.0 ± 1.4	5.1 ± 0.8			

Respiratory and hemodynamic parameters were assessed in anesthetized and surfactant-depleted pigs which underwent either an automated closed-loop protective low tidal volume (6 ml/kg BW LV_T_) and tabular PEEP ventilation strategy (group A) or non-automated injurious high tidal volume ventilation (HV_T_) with high V_T_ of 17 ml/kg BW and PEEP 2 cmH_2_O (group B) during ventilation phase 1. Ventilation phase 2 consisted of automated LV_T_ ventilation in both groups (*N* = 6 each). Two animals died after injurious HV_T_ and RM 2, i.e., Group B, *N* = 4 during ventilation phase 2. For experimental protocol, see Fig. 1 and “Methods” Section. Means ± SD; *p*-values for group effects (inter-group) and/or time effects (intra-group) were calculated using two-way ANOVA-type statistic using *nparLD package.* For more statistical details refer to main text. PIP: peak inspiratory pressure, *PEEP* positive end-expiratory pressure, *ΔP* driving pressure, *V*_*T*_ tidal volume, *RR* respiratory rate, *M*_*P*_ mechanical power, *C*_*rs*_ dynamic respiratory system compliance, *P*_*a*_*O*_*2*_ arterial partial pressure of oxygen, *P*_*a*_*CO*_*2*_ arterial partial pressure of carbon dioxide, *mPAP* mean pulmonary artery pressure, *PVR* pulmonary vascular resistance, *CO* cardiac output

**Fig. 6:**
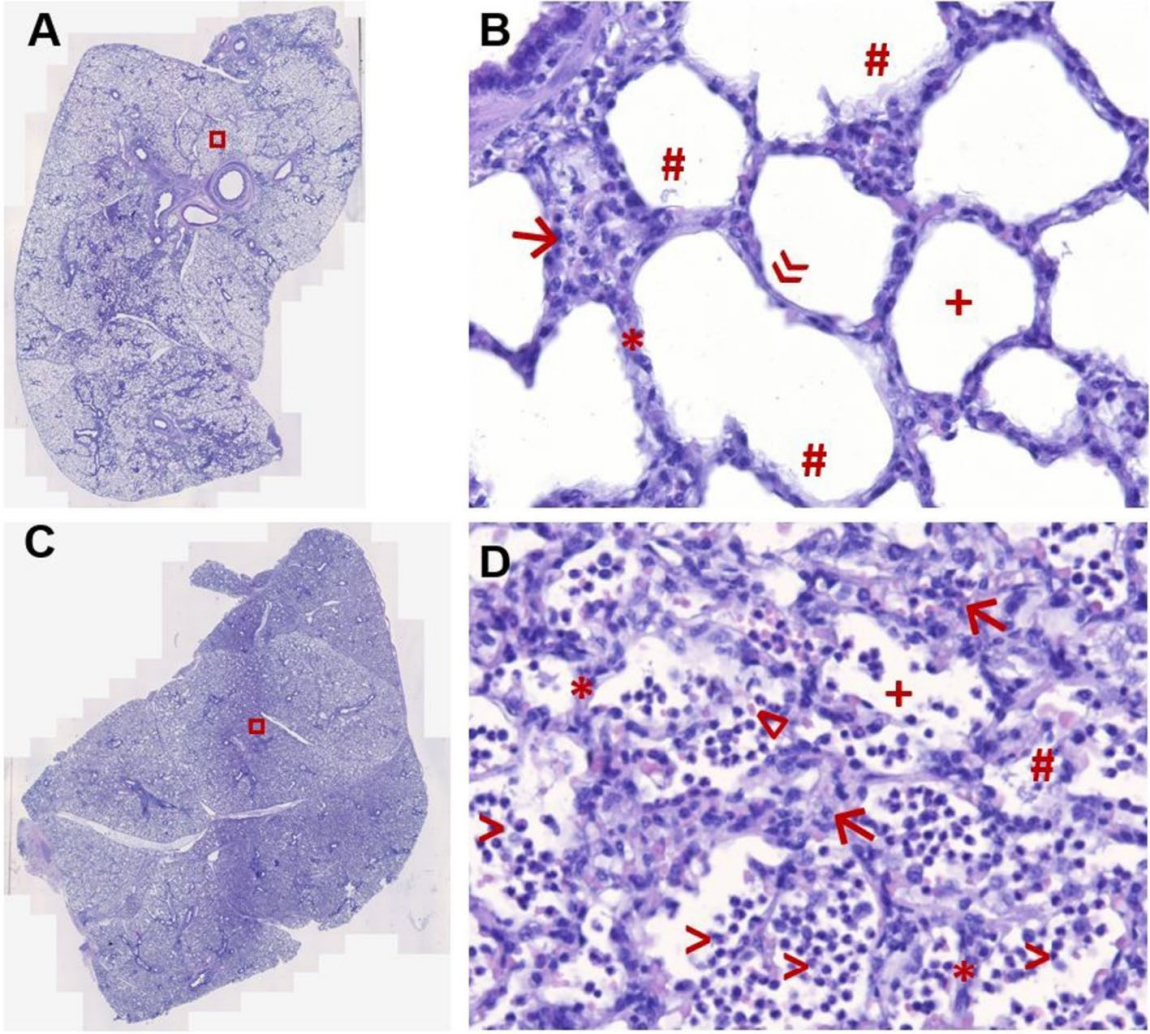
Histopathology. Representative tissue sections stained with hematoxylin/eosin of the right lower lung lobe of pigs after lavage-induced surfactant depletion and subsequent protective **A**, **B** or injurious **C**, **D** mechanical ventilation. High magnification fields (**B**, **D**, × 400) correspond to red box on low magnification field (**A**, **C**, × 100). Note the condensed histoarchitecture and signs of diffuse alveolar damage present after lavage and injurious ventilation (**C**, **D**), including septal thickening (*), massive interstitial ( →) and intra-alveolar ( >) infiltration of neutrophils, intra-alveolar erythrocytes (∆), protein strands (#), and disruption of the alveolar integrity ( +). In contrast, more aerated surface area with preserved alveolar architecture ( +), identification of type I pneumocytes (»), and less alveolar damage is present after lavage and protective ventilation (**A**, **B**). Septal thickening, neutrophil infiltration, and intra-alveolar protein strands occurred to a much lower extent

**Fig. 7:**
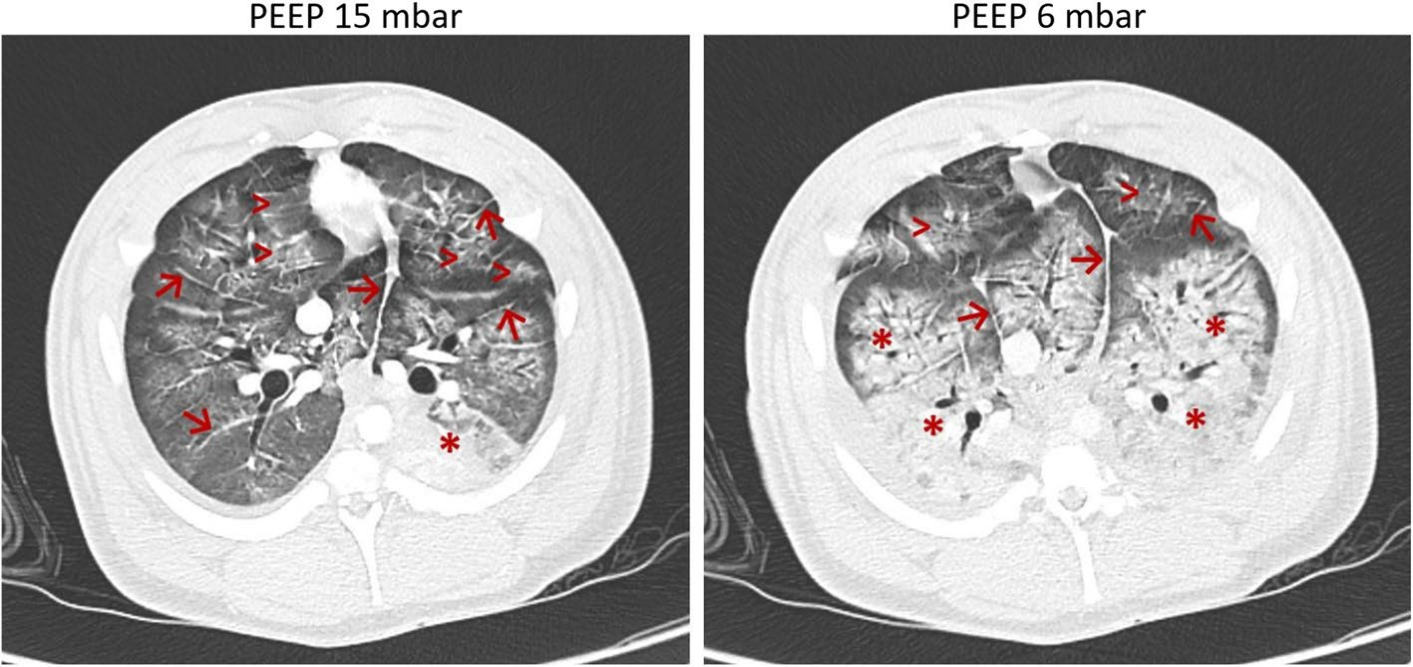
Computed tomography. Representative computed tomography (CT) images of the basal lungs segments of a pig following lavage-induced surfactant depletion and 3 h of injurious ventilation. Images were taken during ventilation with PEEP of 15 vs. 6 mbar and a *V*_*T*_ of 6 mL/kg body weight. Ground glass opacities ( >), interlobar and intralobular septal thickening (→) are representative of the severity of lung injury. Note the significant increase of atelectatic regions (*) as a sign of derecruitment in the dependent lung areas upon PEEP reduction

This model of lavage-induced surfactant depletion and injurious ventilation caused significant histopathological damage (Fig. 6) and CT abnormalities (Fig. 7) typical of ARDS.

### Identification of maximum C_rs_ and oxygenation

Decremental PEEP trials revealed (i) that the PEEP levels producing either maximum C_rs_ or maximum P_a_O_2_ were consistently disparate within each group; (ii) that these “best PEEP” levels were different between the two groups, and (iii) that the inter-group difference of “best oxygenation PEEP” increased progressively over time (Fig. 4, Additional file 1: Table S3–S5): in the group receiving lavage and protective ventilation, PEEP was 16, 14, and 16 cmH_2_O to achieve maximum P_a_O_2_ of 514 ± 49, 508 ± 90, and 563 ± 83 mmHg, respectively, after PEEP trials 1, 2 and 3. At the same time, “best PEEP” to achieve maximum C_rs_ of 26.0 ± 3.4, 29.7 ± 8.0, and 35.5 ± 10.3 ml/cmH_2_O was 12 cmH_2_O, consistently, after all three PEEP trials. In the group receiving lavage and injurious ventilation, PEEP was 20, 18, and 22 cmH_2_O to achieve maximum P_a_O_2_ of 467 ± 55, 346 ± 62, and 334 ± 148 mmHg, respectively, after PEEP trials 1, 2 and 3. At the same time, “best PEEP” to achieve maximum C_rs_ of 24.3 ± 6.2, 28.7 ± 4.7, and 24.8 ± 5.3 ml/cmH_2_O was 16 cmH_2_O, consistently, after all three PEEP trials.

### Invasiveness of ventilation and mechanical power (*M*_*P*_) calculation

Figure 5 depicts the different levels of *V*_*T*_ and *M*_*P*_ applied during protective versus injurious ventilation, caused by different settings of *V*_*T*_ (Fig. 5), PEEP and RR (Table 2), according to the protocol. Automatic adjustment of these settings occurred during automated protective closed-loop ventilation. Note that after resumption of automated protective ventilation during the final 3 h (ventilation phase 2), PEEP (Table 2) and M_P_ (Fig. 5) remain higher in the group coming off injurious ventilation as compared to the group having received protective ventilation only. However, no such difference is reflected in the M_P_, when calculated immediately after RM 2 and 3 at arbitrary standard conditions of PEEP 15 cmH_2_O and V_T_ 6 ml/kg BW (Table 1).

### Hemodynamics

Mean PAP (mPAP), pulmonary vascular resistance (PVR) and cardiac output (CO) were comparable at baseline. All of these parameters increased after lavage and RM (Table 1). Consistently, a significant group effect was found over the course of the PVR during protective and injurious ventilation periods and after RMs (Tables 1 and 2), resulting in higher values in the injurious as compared to the protective ventilation group.

Two animals died of hypoxemia and cardio-circulatory decompensation during the PEEP trial following RM 2 in the injurious ventilation group.

## Discussion

### Summary statement

Models of acute lung injury induced by pulmonary lavages are constrained by their short-lived and incomplete imitation of the pulmonary function characteristics of ARDS, because of the ease of re-opening surfactant-depleted alveoli through application of high peak and positive end-expiratory pressures, which then cause prompt improvements in arterial oxygenation. We found that transitory injurious high tidal–low PEEP ventilation of surfactant-depleted lungs produces a type of sustained lung injury characterized by low recruitability, high levels of titrated PEEP, and oxygenation impairment consistent with mild-to-moderate ARDS during protective ventilation. This model rectifies the constraints of single-hit models employing either only high *V*_*T*_ ventilation or only surfactant-depletion and provides a more realistic means to study the clinical effect of protective ventilation strategies, and can be used to verify the functionality of automated protective closed-loop ventilation.

### Methodological considerations

Recently, significant advances have been made in the engineering of automated physiological closed-loop control systems of mechanical ventilation [21]. These systems shall ensure optimal gas exchange and prevention of VILI. In this context, investigators must pay special attention to choosing the model best suited to test the clinical performance of these systems [22]. The clinical relevance of simple VILI models, in which mechanical ventilation is the sole method used to generate injury, is sometimes questioned as these models typically require very high *V*_*T*_ (up to 30–40 ml/kg BW which is beyond clinical practice) before the shear stresses are high enough to cause injury of an otherwise healthy lung [23, 24]. Lung overinflation as a result of high *V*_*T*_, can cause significant changes in the composition and function of surfactant, as surfactant is squeezed out of the alveolus, which in turn contributes to alveolar instability, cyclic opening and closing, and concomitant inflammation [25]. In contrast, in an injured lung, where the synthesis, distribution and function of surfactant are already impaired, such as after lavage, acid aspiration or secondary to pneumonia and ARDS, injurious shear stress due to overstretching and atelectrauma can occur at much lower *V*_*T*_. Therefore, the intention of this investigation was to characterize a model which combines pulmonary lavage with injurious ventilation to induce a type of lung injury that includes the pathophysiological features of both surfactant depletion or dysfunction, and mechanical stress and strain from ventilation. This model should emulate the clinical situation of patients with ARDS. Importantly, after induction of injury the model was evaluated during standard protective ventilation conditions, in this case with automated closed-loop ventilation. Since this model was destined for the testing of automated protective ventilation strategies, the evaluation of the model was focused around (I) the recruitability of the injured lung; (II) the identification of the PEEP levels producing either the maximum P_a_O_2_ or the maximum *C*_rs_ (“best PEEP”) and III) the M_P_ resulting from automated protective ventilation under these conditions:

### Recruitability

Recruitment of atelectatic lung regions and mechanical ventilation with a PEEP level targeting the prevention of end-expiratory alveolar collapse to abrogate the high shear forces present during cyclic recruitment/decruitment of alveoli, is a pathophysiologically sound “open lung” concept [26]. However, it is important to note that evidence from large clinical trials shows that the routine use of higher PEEP and/or RMs did not reduce mortality in unselected patients with ARDS [27, 28]. In particular, pulmonary recruitability with prompt and large improvements of gas exchange is not a characteristic of the majority of patients with ARDS [10]. In line with these findings, our model of combined lavage and injurious ventilation generated limited recruitability and long-lasting impairment of gas exchange and therefore emulates the pulmonary function characteristics of ARDS better than lavage or VILI alone. Moreover, compared to pulmonary lavage alone, addition of injurious ventilation was associated with higher PVR and higher ∆*P* during and beyond the injurious ventilation phase (Table 2). This difference persisted after RMs 2 and 3, and was associated with higher pulmonary artery pressures and greater hypercapnia than in the group with pulmonary lavage and protective ventilation (Table 1). Therefore, beyond the limited recruitability and sustained hypoxemia, our model also emulates the clinical characteristics of hypercapnic lung failure with pulmonary hypertension, which both have distinct biological and physiological effects in ARDS [29]. In addition, our data show that the impairment of oxygenation and pulmonary compliance, induced by pulmonary lavage alone, progressively faded while the animals received automated protective ventilation with low V_T_ and tabular PEEP (ARDSNet strategy): after 6 h of protective ventilation a P_a_O_2_/F_I_O_2_ ratio of 445±144 mmHg at PEEP 6 cmH_2_O no longer indicated the presence of ALI or ARDS in this group. Consequently, simple models of lavage-induced surfactant depletion may tend to overestimate the therapeutic effects of recruitment maneuvers and lung-protective ventilation strategies, and therefore are not ideal to evaluate the functionality of automated closed-loop and other protective ventilation strategies.

### PEEP titration

Application of PEEP is currently the primary strategy to minimize dynamic strain caused by alveolar recruitment/derecruitment in mechanically ventilated patients with ARDS. Multiple methods have been tested and been reviewed elsewhere [30]. The concept of “best PEEP” was coined by Suter et al. and defined as the level of maximum oxygen delivery (DO_2_) in patients with ARDS [31]. They found that the individual “best PEEP (DO_2_)” was associated with the maximal respiratory system compliance. This is in contrast to our data, where maximal DO_2_ (Additional file 1: Tables S3–S5) was not associated with PEEP values at the maximum of C_rs_ (Fig. 4). Of importance, Suter et al. performed incremental PEEP trials, whereas, in the present model, we performed decremental PEEP trials. Due to potential impairment of venous return and compression of small alveolar vessels, PEEP can affect DO_2_ as much as it affects cardiac output and right ventricular stroke volume [32, 33]. Likewise, when PEEP is reduced, such as in a decremental PEEP trial, cardiac output will increase due to enhanced venous return and reduced alveolar vascular compression. In line with these pathophysiological considerations, we observed cardiac output increasing and PVR decreasing during the decremental PEEP trials in our model. Finally, our data confirm that the PEEP levels required for maximum P_a_O_2_ are consistently higher than the PEEP required to reach maximum *C*_rs_, which is a characteristic feature of ARDS in patients [31]. In our model, the difference between the two “best PEEP” levels of maximum P_a_O_2_ vs. *C*_rs_ after pulmonary lavage (Δ = 4 cmH_2_O in PEEP trial 1) was most pronounced after high *V*_*T*_ ventilation had induced additional injury, and protective ventilation had been resumed (Δ = 6 cmH_2_O in PEEP trial 3). The general concept and provision for these disparate PEEP levels is of high relevance in the clinical setting: while low V_T_ is almost consistently associated with lower P_a_O_2_ than more invasive ventilation strategies [15], the target of maximizing oxygenation through high PEEP, categorical recruitment maneuvers, and higher *V*_*T*_ can result in excessive mortality [27, 28].

### Mechanical power

Recently, VILI has been related to the mechanical power (*M*_*P*_) of ventilation [34]. M_P_ represents the amount of energy per unit of time transferred from the ventilator to the respiratory system and lung tissue [17, 35]. M_P_ and can be calculated as the product of the respiratory rate, *V*_*T*_ and the sum of PEEP and ΔP [18]. In patients receiving invasive ventilation, high M_P_ of ventilation is independently associated with higher in-hospital mortality and several other outcomes [36–38]. In our model, injurious ventilation following pulmonary lavage exposed the lungs to higher M_P_ than protective ventilation, and this is mainly caused by the higher magnitude of the V_T_. Of note, once protective ventilation was resumed, and therefore *V*_*T*_ was ~ 6 ml/kg BW in both groups, M_P_ remained higher in the group that had previously received lavage and injurious ventilation as compared to the group with lavage and protective ventilation (Fig. 5). The higher M_P_ reflects the extent of lung injury caused by injurious ventilation, and can be attributed to the higher peak, end-expiratory and driving pressures that ensued when the automated protective ventilation algorithm (adherent to the rules of the ARDSNet protective ventilation strategy) was applied subsequent to injurious ventilation (Table 2). Only after a RM at 3 h after the resumption of protective ventilation and when lung mechanics were assessed under arbitrary uniform ventilation conditions (*V*_*T*_ 6 ml/kg BW, PEEP 15 cmH_2_O), M_P_ reached a comparable level in both groups (Table 1). While these findings challenge the notion that M_P_ may reflect injury severity, they support that high M_P_ can be reflective of injurious ventilation. However, to estimate the clinical relevance of *M*_*P*_, i.e., the “intensity” of mechanical ventilation—which is outside the aims and scope of the present model—it should be normalized to the size of the ventilated pulmonary surface area [34]. Taking into account that M_P_ reflects the synergy of various different ventilator parameters which may predispose to lung injury associated with mechanical ventilation, further studies are needed to clarify how *M*_*P*_ should guide the choice of ventilator settings.

## Limitations

Since the primary focus of our work was to evaluate the pulmonary function characteristics of the model, and its applicability to serve as a test model for automated protective ventilation strategies, edema formation and biomarkers of inflammation were not evaluated. In addition, since decremental PEEP trials were performed at constant Δ*P*, we cannot exclude that fluctuations of *V*_*T*_ had an impact on the assessment of C_rs_ and oxygenation. Finally, when interpreting pulmonary artery pressure, PVR, and cardiac output obtained during PEEP trials, it must be taken into account that significant hypercapnia occurred at high PEEP levels (Additional file 1: Table S3–S5).

## Conclusion

In summary, we demonstrate that addition of transitory injurious ventilation to surfactant-depleted lungs and subsequent resumption of protective ventilation causes prolonged acute lung injury with low recruitability yielding high titrated PEEP levels. Mimicking the characteristics of lung function and oxygenation impairment of mild-to-moderate ARDS, this porcine model rectifies the constraints of simpler models and provides a more realistic means that may facilitate the translation of experimental research on mechanical ventilation strategies, including closed-loop automated protective ventilation systems. Such systems help advance the evolution of sophisticated personalized protective ventilation strategies in the future.

## Supplementary Information

**Additional file 1: Table S1.** Relative treatment effect values of respiratory and hemodynamic parameters of Tab. 1. **Table S2.** Relative treatment effect values of respiratory and hemodynamic parameters during low and high tidal volume ventilation. **Table S3–S5.** PEEP-Titration.

## Data Availability

The datasets used and/or analyzed during the current study are included within the article are available from the corresponding author on reasonable request.

## Abbreviations

ALI: Acute lung injury
ARDS: Acute respiratory distress syndrome
ARDSNet: Acute Respiratory Distress Syndrome Network
BW: Body weight
C_rs_: Dynamic compliance of the respiratory system
CO: Cardiac output
DO_2_: Delivery of oxygen
ΔP: Pressure difference, the “delta pressure” = driving pressure = PIP − PEEP
F_I_O_2_: Fraction of inspired oxygen
HV_T_: High tidal volume
I:E: Inspiratory-to-expiratory time ratio
LV_T_: Low tidal volume
M_P_: Mechanical power
mPAP: Mean pulmonary artery pressure
P_a_O_2_: Arterial partial pressure of oxygen
P_a_CO_2_: Arterial partial pressure of carbon dioxide
PEEP: Positive end-expiratory pressure
PIP: Peak inspiratory pressure
PVR: Pulmonary vascular resistance
pH_a_: Arterial pH
RM: Recruitment maneuver
RR: Respiratory rate
SpO_2_: Peripheral oxyhemoglobin saturation
V-V ECMO: Veno-venous extracorporeal membrane oxygenation
VILI: Ventilator-induced lung injury
V_T_: Tidal volume
