# Supplementary material for: Induction of severe hypoxemia and low lung recruitability for the evaluation of therapeutic ventilation strategies: a translational model of combined surfactant-depletion and ventilator-induced lung injury
Source: Intensive Care Med Exp. 2022 Jul 29;10:32. doi: 10.1186/s40635-022-00456-5 (PMC9334469; doi:10.1186/s40635-022-00456-5)
Supplement: Supplementary file 1 — Additional file 1: Table S1. Relative treatment effect values of respiratory and hemodynamic parameters of Tab. 1. Table S2. Relative treatment effect values of respiratory and hemodynamic parameters during low and high tidal volume ventilation. Table S3–S5. PEEP-Titration. [file 40635_2022_456_MOESM1_ESM.pdf]

Supplementary Material

Tab. S1: Relative treatment effect (RTE) values of respiratory and hemodynamic parameters of Tab. 1

	Group	Baseline	after Lavage	+ RM 1 *after Lavage*	+ RM 2 *after 3h LV_T vs. HV_T*	+ RM 3 *after 3h LV_T*
**PIP** [cmH_2O]	A	0.121	0.579	0.642	0.549	0.443
	B	0.092	0.662	0.672	0.785	0.433
**PEEP** [cmH_2O]	A	0.263	0.138	0.621	0.718	0.718
	B	0.289	0.138	0.718	0.816	0.621
**△P** [cmH_2O]	A	0.341	0.841	0.509	0.396	0.271
	B	0.273	0.866	0.561	0.626	0.257
**M_p** [J/min]	A	0.757	0.391	0.506	0.427	0.297
	B	0.723	0.546	0.516	0.524	0.220
**C_{rs}** [ml/cmH_2O]	A	0.751	0.137	0.441	0.542	0.731
	B	0.845	0.103	0.447	0.412	0.636
**P_aO_2** [mmHg]	A	0.841	0.178	0.632	0.591	0.746
	B	0.858	0.164	0.455	0.196	0.351
**P_aCO_2** [mmHg]	A	0.117	0.430	0.444	0.668	0.633
	B	0.072	0.532	0.488	0.759	0.928
**mPAP** [mmHg]	A	0.170	0.599	0.484	0.513	0.430
	B	0.079	0.730	0.461	0.851	0.776
**PVR** [$dyn \cdot sec \cdot cm^{-5}$]	A	0.361	/	0.607	0.443	0.288
	B	0.292	/	0.798	0.851	0.488
**CO** [L/min]	A	0.292	/	0.407	0.591	0.679
	B	0.439	/	0.288	0.637	0.813

Experimental protocol see Fig. 1. Means ± SD of parameters in Tab. 1 were analyzed using two-way ANOVA-type nparLD package. The relative treatment effect (RTE) characterizes a tendency of a randomly selected observation in a subgroup to take a smaller or larger value than a randomly selected observation from the entire data set. The RTE of a subgroup can take values between 0 and 1. An RTE = 0.5 indicates a probability of 50 % that a randomly selected value from the subgroup takes on a larger value than a randomly selected value from the entire data set, i.e., no tendency towards smaller or larger values in the subgroup. Consequently, an RTE value < 0.5 suggests a < 50 % probability that a randomly selected value from a subgroup is larger than a randomly selected value from the entire data set, i.e., a tendency for the subgroup to take smaller values and vice versa. RTE values are mapped on

a color scale where 0 is white and 1 is dark red. PIP: peak inspiratory pressure; PEEP: positive end-expiratory pressure; ΔP: driving pressure; MP: mechanical power; Crs: dynamic respiratory system compliance; PaO2: arterial partial pressure of oxygen; PaCO2: arterial partial pressure of carbon dioxide; mPAP: mean pulmonary artery pressure; PVR: pulmonary vascular resistance; CO: cardiac output

Tab. S2: Relative treatment effect values of respiratory and hemodynamic parameters during low and high tidal volume ventilation

	Group	Ventilation Phase 1			Ventilation Phase 2		
		1 h	2 h	3 h	1 h	2 h	3 h
**PIP** [cmH_2O]	A	0.2801	0.2500	0.2199	0.2778	0.3361	0.3083
	B	0.7338	0.7454	0.7708	0.8583	0.7667	0.7417
**PEEP** [cmH_2O]	A	0.7755	0.7222	0.7523	0.3000	0.3778	0.3333
	B	0.2500	0.2500	0.2500	0.8542	0.7167	0.6625
**△P** [cmH_2O]	A	0.2523	0.2569	0.2407	0.3694	0.3639	0.4056
	B	0.7338	0.7454	0.7708	0.6958	0.6333	0.7125
**V_t** [ml/kg BW]	A	0.1968	0.2384	0.3148	0.3972	0.3861	0.4278
	B	0.7500	0.7500	0.7500	0.6458	0.6542	0.6333
**RR** [x/min]	A	0.7407	0.7546	0.7546	0.3333	0.4444	0.5222
	B	0.2500	0.2500	0.2500	0.6000	0.6000	0.6000
**M_p** [J/min]	A	0.2338	0.2361	0.3449	0.2472	0.3250	0.3444
	B	0.7106	0.7199	0.7546	0.8625	0.7458	0.7667
**C_{rs}** [ml/cmH_2O]	A	0.3866	0.4560	0.5093	0.5083	0.5083	0.5472
	B	0.6389	0.5046	0.5046	0.4250	0.4833	0.4958
**P_aO_2** [mmHg]	A	0.4491	0.3565	0.3380	0.3621	0.3764	0.4828
	B	0.8542	0.5671	0.4352	0.7241	0.7328	0.4569
**PaCO2** [mmHg]	A	0.5457	0.5810	0.5190	0.4023	0.4655	0.4276
	B	0.3667	0.4190	0.5762	0.6509	0.5474	0.5905
**mPAP** [mmHg]	A	0.5069	0.5023	0.3773	0.4611	0.4611	0.3389
	B	0.3866	0.4745	0.7523	0.6417	0.5833	0.6333
**PVR** [$dyn\cdot sec\cdot cm^{-5}$]	A	0.4120	0.3657	0.2870	0.4333	0.4056	0.3000
	B	0.5602	0.6574	0.7176	0.7583	0.7083	0.5750
**CO** [L/min]	A	0.5903	0.6551	0.6574	0.5222	0.6639	0.6472
	B	0.3032	0.3333	0.4606	0.3038	0.3167	0.3750

Experimental protocol see Fig. 1. Means ± SD of parameters in Tab. 2 were analyzed using two-way ANOVA-type *nparLD package*. Abbreviations see Tab. 2. The relative treatment effect (RTE) characterizes a tendency of a randomly selected observation in a subgroup/at an individual time point to take a smaller or larger value than a randomly selected observation from the entire data set. RTE values are mapped on a color scale where 0 is white and 1 is dark red. Detailed explanation of RTE see method section in the main text or legend to Tab. S1. PIP: peak inspiratory pressure; PEEP: positive end-expiratory pressure; ΔP: driving pressure; V_T: tidal volume; RR: respiratory rate; M_P: mechanical power; C_{rs}: dynamic respiratory system compliance; P_aO_2: arterial partial pressure of oxygen; P_aCO_2: arterial partial

pressure of carbon dioxide; mPAP: mean pulmonary artery pressure; PVR: pulmonary vascular resistance; CO: cardiac output

Tab. S3: PEEP trial 1

	Group	PEEP - Trial 1									
		PEEP 24	PEEP 22	PEEP 20	PEEP 18	PEEP 16	PEEP 14	PEEP 12	PEEP 10	PEEP 8	PEEP 6
**PIP** [cmH_2O]	A	39.2 ± 0.8	37.5 ± 0.6	35.5 ± 0.6	33.8 ± 0.4	31.8 ± 0.4	29.8 ± 0.4	27.8 ± 0.4	26.0 ± 0.6	23.7 ± 0.5	22.0 ± 0.0
	B	39.2 ± 0.4	37.7 ± 0.5	35.5 ± 0.6	33.7 ± 0.5	31.8 ± 0.4	29.8 ± 0.4	27.8 ± 0.4	25.8 ± 0.4	23.8 ± 0.5	21.6 ± 0.6
**PEEP** [cmH_2O]	A	24.0 ± 0.0	22.0 ± 0.0	20.0 ± 0.0	18.0 ± 0.0	16.0 ± 0.0	14.0 ± 0.0	12.0 ± 0.0	10.0 ± 0.0	8.2 ± 0.4	6.2 ± 0.4
	B	24.0 ± 0.0	22.0 ± 0.0	20.0 ± 0.0	18.2 ± 0.4	16.0 ± 0.0	14.0 ± 0.0	12.2 ± 0.4	10.0 ± 0.0	8.2 ± 0.5	6.0 ± 0.0
**ΔP** [cmH_2O]	A	15.2 ± 0.8	15.5 ± 0.6	15.5 ± 0.6	15.8 ± 0.4	15.8 ± 0.4	15.8 ± 0.4	15.8 ± 0.4	16.0 ± 0.6	15.7 ± 0.5	16.0 ± 0.0
	B	15.2 ± 0.4	15.7 ± 0.5	15.5 ± 0.6	15.7 ± 0.5	15.8 ± 0.4	15.8 ± 0.4	15.8 ± 0.4	15.8 ± 0.4	15.8 ± 0.5	15.6 ± 0.6
**C_{rs}** [ml/cmH_2O]	A	12.2 ± 1.2	15.3 ± 0.8	18.7 ± 1.4	21.5 ± 1.2	24.2 ± 1.9	25.5 ± 2.5	26.0 ± 3.4	24.3 ± 3.7	21.5 ± 3.6	18.5 ± 3.6
	B	13.0 ± 3.3	17.5 ± 3.0	21.0 ± 3.2	23.3 ± 4.6	24.3 ± 6.2	24.3 ± 7.1	22.7 ± 7.1	20.0 ± 7.1	18.8 ± 5.1	14.6 ± 4.6
**P_aO_2** [mmHg]	A	474 ± 76	484 ± 63	480 ± 50	493 ± 40	514 ± 49	485 ± 42	441 ± 60	389 ± 92	280 ± 123	219 ± 133
	B	384 ± 144	445 ± 78	467 ± 55	462 ± 66	411 ± 130	381 ± 129	332 ± 147	260 ± 143	212 ± 122	137 ± 72
**P_aCO_2** [mmHg]	A	122 ± 21	120 ± 20	101 ± 14	85 ± 13	74 ± 11	68 ± 11	65 ± 11	68 ± 14	76 ± 18	89 ± 24
	B	109 ± 26	101 ± 30	85 ± 24	74 ± 21	72 ± 27	73 ± 29	77 ± 35	87 ± 40	84 ± 30	105 ± 39
**mPAP** [mmHg]	A	25 ± 3	26 ± 2	24 ± 3	23 ± 4	22 ± 5	21 ± 4	21 ± 4	21 ± 5	22 ± 6	24 ± 5
	B	27 ± 3	26 ± 2	24 ± 2	23 ± 2	23 ± 3	23 ± 5	24 ± 7	27 ± 9	28 ± 5	32 ± 6
**PVR** [$dyn \cdot sec \cdot cm^{-5}$]	A	320 ± 76	318 ± 86	247 ± 64	226 ± 30	214 ± 100	198 ± 75	182 ± 57	162 ± 38	175 ± 26	202 ± 42
	B	394 ± 119	335 ± 90	272 ± 82	271 ± 74	274 ± 86	276 ± 87	289 ± 101	334 ± 164	316 ± 109	381 ± 76
**CO** [L/min]	A	3.7 ± 1.3	3.9 ± 1.3	4.4 ± 1.2	4.2 ± 1.5	4.3 ± 1.8	4.6 ± 1.7	4.7 ± 2.1	5.1 ± 2.3	5.4 ± 2.6	5.9 ± 2.8
	B	3.3 ± 1.2	3.5 ± 1.1	3.6 ± 1.0	3.4 ± 0.7	3.4 ± 1.0	3.4 ± 0.9	3.7 ± 1.1	4.0 ± 1.1	4.8 ± 2.1	4.7 ± 1.4
**S_pO_2** [%]	A	99 ± 2	99 ± 2	100 ± 1	99 ± 2	99 ± 2	100 ± 1	99 ± 2	100 ± 1	100 ± 1	99 ± 2
	B	98 ± 3	99 ± 3	99 ± 2	99 ± 1	99 ± 3	98 ± 5	96 ± 9	92 ± 18	97 ± 6	91 ± 11
**Hb** [g/dl]	A	9.1 ± 1.6	9.5 ± 1.6	9.4 ± 1.5	9.4 ± 1.6	9.4 ± 1.7	9.4 ± 1.6	9.4 ± 1.8	9.4 ± 1.9	9.3 ± 1.9	9.5 ± 1.9
	B	9.2 ± 0.5	9.4 ± 0.3	9.4 ± 0.5	9.4 ± 0.6	9.4 ± 0.8	9.4 ± 0.6	9.3 ± 0.6	9.2 ± 0.6	9.1 ± 0.5	9.4 ± 0.6
**C_aO_2** [ml/dl]	A	13.8 ± 2.1	14.3 ± 2.0	14.1 ± 2.1	14.2 ± 2.1	14.2 ± 2.2	14.2 ± 2.1	14.0 ± 2.4	13.8 ± 2.5	13.4 ± 2.5	13.2 ± 2.7
	B	13.2 ± 1.0	14.0 ± 0.4	14.0 ± 0.6	14.0 ± 0.8	13.8 ± 1.0	13.7 ± 0.9	13.2 ± 1.1	12.6 ± 1.5	12.5 ± 0.7	11.8 ± 0.8
**DO_2** [ml/min]	A	503.5 ± 171.8	555.0 ± 176.9	617.7 ± 180.5	593.2 ± 232.0	614.6 ± 249.1	641.1 ± 230.7	659.2 ± 284.4	712.2 ± 326.3	712.2 ± 333.4	765.8 ± 336.2
	B	442.1 ± 169.8	491.7 ± 150.1	503.0 ± 126.4	468.5 ± 91.2	459.7 ± 133.1	464.0 ± 116.1	490.7 ± 139.7	499.7 ± 161.4	599.3 ± 248.9	551.2 ± 138.3

Table depicts parameters of respiratory mechanics, pulmonary function, hemodynamics and oxygen delivery during PEEP trial 1 in anesthetized pigs which underwent lavage-induced surfactant depletion and a pulmonary recruitment maneuver (RM). The PEEP trial consisted of decrements of 2 from 24 to 6 cmH_2O of PEEP and was recorded immediately after the RM. Groups were then destined to undergo two different ventilation strategies. Group A, N=6: continuous automated protective low tidal volume ventilation (LV_T). Group B, N=6: Three hours of injurious high tidal volume ventilation (HV_T) prior to PEEP trial 2 (see Tab. S4), resumption of protective low tidal volume ventilation for three hours prior to PEEP trial 3 (see Tab. S5). Means ± SD. Maximum means of Crs, PaO_2 and DO_2 per group are underscored in red to facilitate retrieval. Crs and PaO_2 are also depicted in Fig 4. Experimental protocol see Fig. 1 and methods section. PIP: peak inspiratory pressure; PEEP: positive end-expiratory pressure; ΔP: driving pressure; Crs: dynamic respiratory system compliance; PaO_2: arterial partial pressure of oxygen; $PaCO_2$: arterial partial pressure of carbon dioxide; mPAP: mean pulmonary artery pressure; PVR: pulmonary vascular resistance; CO: cardiac output; SpO2: peripheral oxygen saturation; Hb: hemoglobin concentration; CaO_2: arterial oxygen content; DO_2: oxygen delivery

Tab. S4: PEEP trial 2

		PEEP - Trial 2									
	Group	PEEP 24	PEEP 22	PEEP 20	PEEP 18	PEEP 16	PEEP 14	PEEP 12	PEEP 10	PEEP 8	PEEP 6
**PIP** [cmH_2O]	A	39.5 ± 0.8	37.7 ± 0.5	35.8 ± 0.4	33.8 ± 0.4	31.8 ± 0.4	19.7 ± 0.8	27.7 ± 0.8	24.0 ± 4.9	23.7 ± 0.8	21.5 ± 0.8
	B	39.2 ± 0.5	37.6 ± 0.6	35.4 ± 0.6	33.7 ± 0.6	31.3 ± 1.2	29.3 ± 1.2	27.3 ± 0.6	25.5 ± 0.7	23.0 ± 0.0	/
**PEEP** [cmH_2O]	A	24.0 ± 0.0	22.0 ± 0.0	20.2 ± 0.4	18.0 ± 0.0	16.2 ± 0.4	14.2 ± 0.4	12.0 ± 0.0	10.2 ± 0.4	8.3 ± 0.5	6.2 ± 0.4
	B	24.0 ± 0.0	22.0 ± 0.0	20.0 ± 0.0	18.0 ± 0.0	16.0 ± 0.0	14.0 ± 0.0	12.0 ± 0.0	10.0 ± 0.0	8.0 ± 0.0	/
**ΔP** [cmH_2O]	A	15.5 ± 0.8	15.7 ± 0.5	15.8 ± 0.4	15.8 ± 0.4	15.8 ± 0.4	15.7 ± 0.8	15.7 ± 0.8	14.0 ± 4.9	15.7 ± 0.8	15.5 ± 0.8
	B	15.2 ± 0.5	15.6 ± 0.6	15.4 ± 0.6	15.7 ± 0.6	15.3 ± 1.2	15.3 ± 1.2	15.3 ± 0.6	15.5 ± 0.7	15.0 ± 0.0	/
**C_{rs}** [ml/cmH_2O]	A	12.8 ± 5.6	16.2 ± 6.1	19.5 ± 7.0	23.2 ± 8.0	25.8 ± 8.0	28.8 ± 9.1	29.7 ± 8.0	29.0 ± 7.4	27.5 ± 6.7	24.2 ± 5.7
	B	13.8 ± 5.5	16.8 ± 6.6	19.4 ± 7.6	26.7 ± 5.5	28.7 ± 4.7	28.7 ± 3.8	26.0 ± 2.0	20.5 ± 2.1	15.0 ± 0.0	/
**P_aO_2** [mmHg]	A	438 ± 76	466 ± 69	492 ± 82	500 ± 89	506 ± 80	508 ± 90	473 ± 92	407 ± 114	319 ± 134	254 ± 121
	B	170 ± 98	216 ± 132	215 ± 146	346 ± 62	308 ± 92	220 ± 108	100 ± 22	119 ± 61	100 ± 0	/
**P_aCO_2** [mmHg]	A	142 ± 29	144 ± 26	125 ± 23	102 ± 19	85 ± 17	71 ± 17	68 ± 15	63 ± 14	68 ± 17	77 ± 19
	B	130 ± 9	137 ± 31	130 ± 39	93 ± 2	87 ± 6	88 ± 8	97 ± 12	108 ± 1	167 ± 0	/
**mPAP** [mmHg]	A	33 ± 6	30 ± 4	28 ± 4	26 ± 4	24 ± 4	22 ± 6	20 ± 7	22 ± 5	19 ± 6	20 ± 6
	B	35 ± 3	34 ± 7	31 ± 4	28 ± 3	26 ± 1	25 ± 1	25 ± 2	26 ± 1	30 ± 0	/
**PVR** [$dyn \cdot sec \cdot cm^{-5}$]	A	339 ± 157	209 ± 126	270 ± 125	227 ± 98	191 ± 87	183 ± 86	155 ± 97	170 ± 65	120 ± 52	116 ± 63
	B	636 ± 605	422 ± 117	418 ± 190	316 ± 4	262 ± 26	239 ± 18	245 ± 55	211 ± 19	23 ± 0	/
**CO** [L/min]	A	5.2 ± 2.3	5.4 ± 2.0	5.3 ± 1.9	5.2 ± 1.8	5.4 ± 2.0	5.4 ± 1.7	5.0 ± 2.0	5.4 ± 2.1	5.7 ± 2.2	6.7 ± 2.4
	B	5.2 ± 3.5	4.9 ± 1.7	4.7 ± 2.0	4.7 ± 1.3	5.2 ± 1.4	5.3 ± 1.2	5.6 ± 1.6	7.1 ± 1.4	8.0 ± 0.0	/
**S_pO_2** [%]	A	99 ± 1	100 ± 1	100 ± 1	100 ± 1	100 ± 1	100 ± 0	100 ± 0	100 ± 0	100 ± 1	99 ± 2
	B	90 ± 10	89 ± 15	90 ± 15	99 ± 1	100 ± 0	100 ± 0	99 ± 1	90 ± 4	51 ± 0	/
**Hb** [g/dl]	A	9.5 ± 1.1	9.6 ± 1.1	9.4 ± 1.1	9.3 ± 1.3	9.2 ± 1.2	9.3 ± 1.2	9.3 ± 1.2	9.3 ± 1.1	9.3 ± 1.1	9.3 ± 1.0
	B	10.5 ± 0.7	10.4 ± 1.1	10.1 ± 1.3	9.0 ± 0.6	8.7 ± 0.8	8.7 ± 0.8	8.8 ± 0.6	9.1 ± 0.9	8.5 ± 0.0	/
**C_aO_2** [ml/dl]	A	14.1 ± 1.6	14.3 ± 1.5	14.2 ± 1.5	14.0 ± 1.7	14.0 ± 1.7	14.0 ± 1.6	13.9 ± 1.7	13.7 ± 1.5	13.5 ± 1.4	13.1 ± 1.1
	B	13.4 ± 2.0	13.1 ± 1.8	12.6 ± 1.6	13.1 ± 0.9	12.7 ± 1.1	12.2 ± 1.4	11.4 ± 2.0	10.7 ± 1.4	8.0 ± 0.0	/
**DO_2** [ml/min]	A	718.6 ± 269.9	751.7 ± 233.3	727.9 ± 211.7	715.5 ± 195.9	733.4 ± 203.9	739.9 ± 187.8	678.9 ± 238.9	725.6 ± 255.3	766.2 ± 266.2	873.19 ± 290.7
	B	647.0 ± 384.6	589.1 ± 188.6	564.9 ± 236.9	624.3 ± 201.7	663.0 ± 229.3	655.5 ± 222.9	648.5 ± 257.1	748.6 ± 58.0	633.6 ± 0.0	/

Table depicts parameters of respiratory mechanics, pulmonary function, hemodynamics and oxygen delivery during PEEP trial 2 in anesthetized pigs which had undergone lavage-induced surfactant depletion, 3 hours of either low or high tidal volume ventilation and a pulmonary recruitment maneuver (RM). The PEEP trial consisted of decrements of 2 from 24 to 6 cmH_2O of PEEP and was recorded immediately after the RM. Group A, N=6: continuous automated protective low tidal volume ventilation (LV_T). Two animals died after injurious HV_T and RM 2, Group B, N=4: Three hours of injurious high tidal volume ventilation (HV_T) prior to PEEP trial 2 (see Tab. S4), resumption of protective low tidal volume ventilation for three hours prior to PEEP trial 3 (see Tab. S5). Means ± SD. Maximum means of C_{rs}, P_aO_2 and DO_2 per group are underscored in red to facilitate retrieval. Crs and PaO_2 are also depicted in Fig 4. Experimental protocol see Fig. 1 and methods section. PIP: peak inspiratory pressure; PEEP: positive end-expiratory pressure; ΔP: driving pressure; Crs: dynamic respiratory system compliance; PaO_2: arterial partial pressure of oxygen; $PaCO_2$: arterial partial pressure of carbon dioxide; mPAP: mean pulmonary artery pressure; PVR: pulmonary vascular resistance; CO: cardiac output; SpO2: peripheral oxygen saturation; Hb: hemoglobin concentration; CaO_2: arterial oxygen content; DO_2: oxygen delivery

Tab. S5: PEEP trial 3

	Group	PEEP - Trial 3									
		PEEP 24	PEEP 22	PEEP 20	PEEP 18	PEEP 16	PEEP 14	PEEP 12	PEEP 10	PEEP 8	PEEP 6
**PIP** [cmH_2O]	A	39.5 ± 0.8	37.7 ± 0.8	35.7 ± 0.8	33.7 ± 0.8	31.7 ± 0.8	29.7 ± 0.8	27.7 ± 0.8	25.5 ± 0.8	23.7 ± 0.8	21.7 ± 0.8
	B	40.0 ± 0.0	38.0 ± 0.0	36.0 ± 0.0	33.8 ± 0.5	31.8 ± 0.5	29.8 ± 0.5	27.8 ± 0.5	26.0 ± 0.0	24.0 ± 0.0	/
**PEEP** [cmH_2O]	A	24.0 ± 0.0	22.0 ± 0.0	20.0 ± 0.0	18.2 ± 0.4	16.2 ± 0.4	14.3 ± 0.5	12.3 ± 0.5	10.2 ± 0.4	8.2 ± 0.4	6.2 ± 0.4
	B	24.0 ± 0.0	22.0 ± 0.0	20.0 ± 0.0	18.0 ± 0.0	16.0 ± 0.0	14.0 ± 0.0	12.0 ± 0.0	10.0 ± 0.0	8.0 ± 0.0	/
**ΔP** [cmH_2O]	A	15.5 ± 0.8	15.7 ± 0.8	15.7 ± 0.8	15.7 ± 0.8	15.7 ± 0.8	15.7 ± 0.8	15.7 ± 0.8	15.5 ± 0.8	15.7 ± 0.8	15.7 ± 0.8
	B	16.0 ± 0.0	16.0 ± 0.0	16.0 ± 0.0	15.8 ± 0.5	15.8 ± 0.5	15.8 ± 0.5	15.8 ± 0.5	16.0 ± 0.0	16.0 ± 0.0	/
**C_{rs}** [ml/cmH_2O]	A	15.0 ± 6.6	19.3 ± 8.1	23.0 ± 9.0	27.0 ± 9.7	30.8 ± 10.5	33.8 ± 10.7	35.5 ± 10.3	35.5 ± 9.1	33.7 ± 7.8	30.5 ± 6.3
	B	16.3 ± 1.9	19.5 ± 2.5	22.0 ± 3.5	23.5 ± 4.4	24.8 ± 5.3	24.0 ± 5.6	22.0 ± 5.9	21.0 ± 2.8	19.0 ± 0.0	/
**P_aO_2** [mmHg]	A	481 ± 74	495 ± 84	530 ± 94	539 ± 72	563 ± 83	559 ± 71	557 ± 82	534 ± 99	490 ± 127	445 ± 144
	B	322 ± 135	334 ± 148	329 ± 159	313 ± 166	266 ± 164	154 ± 129	102 ± 65	84 ± 37	74 ± 0	/
**P_aCO_2** [mmHg]	A	117 ± 21	115 ± 33	99 ± 32	84 ± 29	71 ± 23	60 ± 18	55 ± 12	53 ± 11	54 ± 11	57 ± 11
	B	138 ± 7	128 ± 14	116 ± 23	112 ± 30	107 ± 38	109 ± 48	123 ± 43	105 ± 1	125 ± 0	/
**mPAP** [mmHg]	A	28 ± 3	26 ± 3	24 ± 2	23 ± 3	23 ± 2	21 ± 3	19 ± 3	18 ± 4	17 ± 4	18 ± 4
	B	33 ± 2	31 ± 4	26 ± 1	25 ± 2	27 ± 1	25 ± 2	26 ± 5	23 ± 4	21 ± 0	/
**PVR** [$dyn \cdot sec \cdot cm^{-5}$]	A	236 ± 79	190 ± 57	160 ± 39	148 ± 67	133 ± 25	118 ± 46	99 ± 45	75 ± 41	67 ± 37	69 ± 32
	B	309 ± 99	291 ± 60	209 ± 93	184 ± 107	204 ± 77	197 ± 94	196 ± 82	140 ± 27	79 ± 0	/
**CO** [L/min]	A	6.0 ± 2.4	6.2 ± 2.0	6.4 ± 2.1	6.4 ± 1.7	6.5 ± 1.7	7.2 ± 2.5	7.1 ± 2.1	8.0 ± 2.4	8.0 ± 2.5	8.4 ± 2.7
	B	5.8 ± 1.6	5.6 ± 0.9	5.9 ± 0.8	6.6 ± 1.1	6.5 ± 0.8	6.5 ± 0.6	6.8 ± 0.7	7.9 ± 0.9	12.2 ± 0.0	/
**S_pO_2** [%]	A	99 ± 2	98 ± 3	98 ± 3	98 ± 3	99 ± 2	99 ± 3	98 ± 4	99 ± 2	99 ± 2	99 ± 2
	B	98 ± 2	99 ± 2	99 ± 2	99 ± 3	98 ± 5	95 ± 9	84 ± 15	82 ± 16	69 ± 0	/
**Hb** [g/dl]	A	9.2 ± 0.9	9.2 ± 0.6	9.1 ± 0.6	9.0 ± 0.7	9.0 ± 0.6	8.9 ± 0.6	8.6 ± 1.0	8.7 ± 1.1	8.6 ± 1.1	8.6 ± 1.2
	B	9.5 ± 1.1	9.2 ± 1.2	9.1 ± 1.3	9.2 ± 1.2	9.2 ± 1.1	9.1 ± 1.2	9.2 ± 1.1	8.5 ± 0.2	8.2 ± 0.0	/
**C_aO_2** [ml/dl]	A	13.9 ± 1.2	13.9 ± 0.8	13.9 ± 0.6	13.9 ± 0.8	13.9 ± 0.6	13.8 ± 0.7	13.3 ± 1.3	13.4 ± 1.4	13.1 ± 1.3	12.9 ± 1.4
	B	12.6 ± 2.4	12.4 ± 2.3	12.5 ± 1.8	13.0 ± 0.7	12.4 ± 0.4	11.0 ± 0.9	9.1 ± 2.1	8.4 ± 3.0	7.7 ± 0.0	/
**DO_2** [ml/min]	A	832.8 ± 357.1	851.3 ± 263.2	931.6 ± 273.9	887.3 ± 235.5	962.6 ± 215.1	1036.2 ± 341.5	924.1 ± 279.3	1051.4 ± 309.4	1070.2 ± 333.2	1112.8 ± 392.5
	B	779.6 ± 195.8	736.3 ± 102.8	772.0 ± 64.3	844.6 ± 107.8	802.0 ± 114.4	716.5 ± 107.4	630.7 ± 190.3	648.9 ± 156.3	930.5 ± 0.0	/

Table depicts parameters of respiratory mechanics, pulmonary function, hemodynamics and oxygen delivery during PEEP trial 3 in anesthetized pigs which had undergone lavage-induced surfactant depletion and mechanical ventilation. The PEEP trial consisted of decrements of 2 from 24 to 6 cmH_2O of PEEP and was recorded immediately after a RM. Group A, N=6: continuous automated protective low tidal volume ventilation (LV_T). Two animals died after injurious HV_T and RM 2, Group B, N=4: Three hours of injurious high tidal volume ventilation (HV_T) prior to PEEP trial 2 (see Tab. S4), resumption of protective low tidal volume ventilation for three hours prior to PEEP trial 3. Means ± SD. Maximum means of C_{rs}, P_aO_2 and DO_2 per group are underscored in red to facilitate retrieval. Crs and PaO_2 are also depicted in Fig 4. Experimental protocol see Fig. 1 and methods section. PIP: peak inspiratory pressure; PEEP: positive end-expiratory pressure; ΔP: driving pressure; Crs: dynamic respiratory system compliance; PaO_2: arterial partial pressure of oxygen; $PaCO_2$: arterial partial pressure of carbon dioxide; mPAP: mean pulmonary artery pressure; PVR: pulmonary vascular resistance; CO: cardiac output; SpO2: peripheral oxygen saturation; Hb: hemoglobin concentration; CaO_2: arterial oxygen content; DO_2: oxygen delivery
